# Repeatability of Perimacular Ganglion Cell Complex Analysis with Spectral-Domain Optical Coherence Tomography

**DOI:** 10.1155/2015/605940

**Published:** 2015-07-01

**Authors:** Dorothy S. K. Ng, Preeti Gupta, Yih Chung Tham, Chye Fong Peck, Tien Yin Wong, Mohammad Kamran Ikram, Carol Y. Cheung

**Affiliations:** ^1^Singapore Eye Research Institute, Singapore National Eye Centre, Singapore 169856; ^2^Department of Ophthalmology, Yong Loo Lin School of Medicine, National University of Singapore, Singapore 117597; ^3^Ophthalmology and Visual Sciences Academic Clinical Programme, Duke-NUS Graduate Medical School, National University of Singapore, Singapore 169857

## Abstract

*Purpose*. To assess the repeatability of spectral-domain optical coherence tomography to measure macular and perimacular ganglion cell complex thicknesses and compare retinal ganglion cell parameters between algorithms. *Methods*. Ninety-two nonglaucomatous eyes from 92 participants underwent macular and perimacular ganglion cell complex thickness measurement using OCT-HS100 Glaucoma 3D algorithm and these measurements were repeated for 34 subjects. All subjects also had macular ganglion cell-inner plexiform layer thickness measured by Cirrus HD-OCT Ganglion Cell Analysis algorithm. Intraclass correlation coefficient and Pearson's correlation analyses were performed. *Results*. Subfields of both macular and perimacular ganglion cell complex thicknesses had high intraclass correlation coefficient values between 0.979 (95% confidence interval [CI]: 0.958–0.989) and 0.981 (95% CI: 0.963, 0.991) and between 0.70 (95% CI: 0.481–0.838) and 0.987 (95% CI: 0.956–0.989), respectively. The overall average ganglion cell complex and macular average ganglion cell-inner plexiform layer thicknesses were strongly correlated (*r* = 0.83,  *P* < 0.001).  *Conclusions*. The assessment of macular and perimacular retinal ganglion cell parameters by OCT-HS100 Glaucoma 3D algorithm is highly repeatable, and strongly correlates to retinal ganglion cell parameters assessed by Ganglion Cell Analysis algorithm. A comprehensive evaluation of retinal ganglion cells may be possible with OCT-HS100.

## 1. Introduction

There is increasing evidence that assessment of macular ganglion cell complex (GCC) by spectral-domain optical coherence tomography (SD-OCT) algorithms is useful for early detection of retinal ganglion cell (RGC) damage in progressive and potentially blinding ophthalmic diseases. Several studies on macular GCC parameters, comprising retinal nerve fiber layer (RNFL), ganglion cell layer (GCL), and inner plexiform layer (IPL) have shown good glaucoma diagnostic ability [1–3] and detected presence of neurodegeneration in early vascular diabetic retinopathy [4, 5].

Established algorithms for RGC assessment such as the GCC algorithm (RTVue-100, Optovue Inc., Fremont, CA) and the Ganglion Cell Analysis (GCA) algorithm (Cirrus HD-OCT, Carl Zeiss Meditec, Inc., Dublin, CA) acquire only macular data [6–9] and do not provide comprehensive assessment of perimacular RGCs and their axonal extensions that comprise the RNFL bundles. The RNFL bundles are retinotopically organized arching above and below the macula to enter the vertical poles of the optic nerve head, and fibers from the macula pass directly to the nasal pole of the optic nerve head [10]. A larger area of RGC assessment that encompasses both macula and perimacula is essential to facilitate visualization of RGC damage and progression pattern, which would potentially be useful for disease monitoring [11]. More recently, the Glaucoma 3D algorithm (OCT-HS100, Canon Inc., Tokyo, Japan) is made available to measure the GCC from the parafovea to the perimacula while simultaneously accounting for the influence of anatomic variation in the RNFL bundles by aligning the centers of the macula and the optic disc on the same axis. As with any new technique, high measurement consistency is of prime importance for reliable data interpretation [12]. In view of the potential clinical application of GCC parameters, we assessed the repeatability of Glaucoma 3D algorithm developed for OCT-HS100 to measure macular and perimacular GCC thickness in normal healthy eyes. Furthermore, we compared RGC parameters between OCT-HS100 and Cirrus HD-OCT.

## 2. Methods

### 2.1. Study Participants

In this cross-sectional observational study, a total of 92 volunteers aged 18 years or older who met the inclusion criteria were recruited at the Singapore Eye Research Institute. All participants underwent a standardized and comprehensive ophthalmic examination, including best corrected visual acuity (BCVA) measurement with logarithmic minimal angle resolution (logMAR) chart, slit lamp biomicroscopy, dilated fundus examination, Goldmann applanation tonometry, and visual field examination. The static refraction was measured using an autorefractor (Canon RK 5 Auto Ref-Keratometer, Canon Inc., Ltd., Tochigi-ken, Japan). Spherical equivalent refraction was calculated as the sum of the spherical value and half of the cylindrical value. Normal healthy eyes were defined as myopia severity < 6 diopters, BCVA of logMAR ≤ 0.3, intraocular pressure < 22 mmHg, clear ocular media, normal functioning status in both eyes, and no history or evidence of macular disease, previous retinal or refractive surgery, neurologic disease, glaucoma, or visual field defect (as defined below). One randomly selected eye was studied in each eligible participant. Written informed consent was obtained from each participant after explanation of the nature and possible consequences of the study. The study adhered to the tenets of the Declaration of Helsinki, and ethics committee approval was obtained from the SingHealth Centralized Institutional Review Board.

### 2.2. Visual Field Examination

Standardized visual field testing was performed with static automated white-on-white threshold perimetry (Humphrey Field Analyzer II, Carl Zeiss Meditec) with the central 24-2, Swedish Interactive Threshold Algorithm Standard program. A visual field was defined as reliable when fixation losses were <20%, and false-positive and false-negative rates were <33%. A field defect was defined as having three or more significant (*P* < 0.05) nonedge contiguous points with at least one point at *P* < 0.01 level on the same side of the horizontal meridian in the pattern deviation probability plot, classified as outside normal limits in the glaucoma hemifield test and confirmed with at least two visual field examinations.

### 2.3. Spectral-Domain Optical Coherence Tomography Imaging

**Figure 1:**
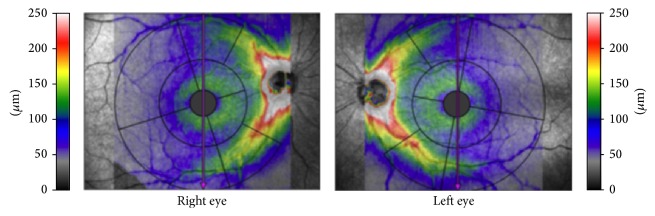
Measurement grids of OCT-HS100 Glaucoma 3D algorithm. The grids are centered on the fovea and rotated in alignment with the centre of the optic disc. Both foveal centre and optic disc centre are aligned on the same axis to account for the influence of anatomic variation in the nerve fiber layer bundles.

**Figure 2:**
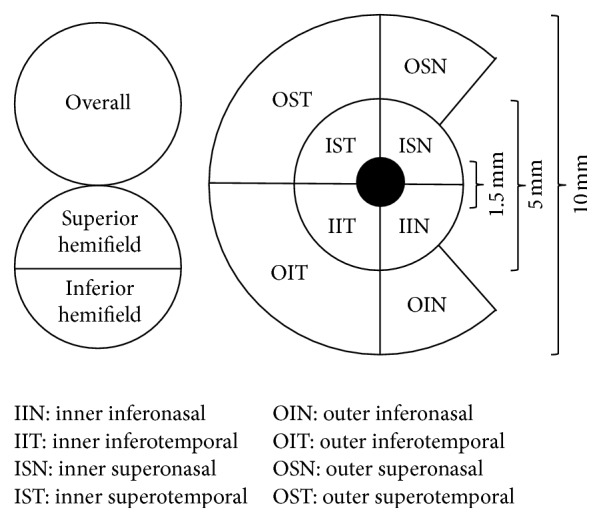
Ganglion cell complex (GCC) thickness map of the right eye in OCT-HS100 Glaucoma 3D algorithm. The area bounded by the inner (1.5 mm) and middle (5 mm) circles forms the inner ring, while the area bounded by the middle (5 mm) and outer (10 mm) circles forms the outer ring. Thicknesses are averaged for the overall measurement, superior and inferior hemifields over the 10 mm diameter circle, and inner (macular GCC) and outer (perimacular GCC) subfields. The section of the nasal outer ring that encroached onto the optic disc and the central 1.5 mm in diameter area are excluded.

The two commercially available SD-OCT devices used in this study were the OCT-HS100 (software version 3.0) and Cirrus HD-OCT (software version 6.0). The OCT-HS100 has a scan speed of 70,000 axial scans per scan with an axial resolution of 3 *μ*m. The Glaucoma 3D scanning protocol incorporated in OCT-HS100 was used to acquire GCC data over a 10 × 10 mm area centered on the fovea with a scan pattern of 128 B-scans, each consisting of 1024 A-scans within a scan time of 1.9 seconds. During the Glaucoma 3D scanning process, the measurement grid was centered on the fovea and automatically rotated to align with the optic disc centre on the same axis to account for the influence of anatomic variation in the RNFL bundles (Figure 1). The inner limiting membrane and the outer boundary of the IPL were automatically segmented by the Glaucoma 3D algorithm; the segmented layer thus led to the measurement of the GCC thickness. The GCC thickness map of the Glaucoma 3D algorithm comprised an inner ring (bounded by 1.5 mm and 5 mm in diameter circles) for macular GCC thickness measurement and an outer ring (bounded by 5 mm and 10 mm in diameter circles) for perimacular GCC thickness measurement (Figure 2). Each ring comprised four subfields (superonasal, inferonasal, inferotemporal, and superotemporal) totaling eight subfields. The central 1.5 mm in diameter area and the nasal part of the outer ring that encroached onto the optic disc were excluded. The computational output of Glaucoma 3D algorithm also computed the GCC thickness for the overall average and superior and inferior hemifields over the 10 mm in diameter circle. We further calculated the macular average GCC thickness, that is, average of the four inner ring subfields.

The Cirrus HD-OCT has a scan speed of 27,000 axial scans per second and an axial resolution of 5 *μ*m. A 6 × 6 mm area centered on the fovea was scanned by the Macular Cube 200 × 200 scanning protocol that has 200 horizontal B-scans, each consisting of 200 A-scans within a scan time of 1.5 seconds [13, 14]. The automated GCA algorithm, incorporated in Cirrus HD-OCT software version 6.0, was used to segment and measure the thickness of ganglion cell-inner plexiform layer (GC-IPL) within an elliptical annulus centered on the fovea based on the three-dimensional data generated from the Macular Cube 200 × 200 protocol. The elliptical annulus has the following dimensions: vertical inner radius and vertical outer radius of 0.5 mm and 2.0 mm, respectively, and horizontal inner radius and horizontal outer radius of 0.6 mm and 2.4 mm, respectively. The size of the inner ring was chosen to exclude the area where the GCL is thin and difficult to detect, whereas the size and shape of the outer ring were chosen to conform closely to the real macular anatomy, where the GCL is thickest in normal eyes [6]. The thickness map of the GCA algorithm contained three sectors on either side of the horizontal midline (superotemporal, superior, superonasal, inferonasal, inferior, and inferotemporal). The outer boundaries of the RNFL and the IPL were segmented by the GCA algorithm; the segmented layer thus led to the measurement of the GC-IPL thickness. The GCA algorithm also computed the macular average GC-IPL thickness. We further calculated the GC-IPL thickness for the superior and inferior hemifields, that is, average of the three sectors on either side of the horizontal midline.

Each study eye was dilated with tropicamide 1% and phenylephrine hydrochloride 2.5%. All 92 participants underwent macular scanning on both SD-OCT devices in the same session by a single operator. The OCT-HS100 Glaucoma 3D scan was repeated for a subset of 34 randomly selected participants within an interval of 30 minutes for the purpose of assessing intrasession repeatability of GCC thickness measurement. OCT scans showing algorithm segmentation error, centration error, signal strength < 6, or artifacts due to eye movements or blinking were excluded from the analysis. Scan of insufficient quality at first attempt was repeated.

### 2.4. Statistical Analysis

The characteristics of participants and GCC parameters were expressed as mean ± standard deviation (SD). The intraclass correlation coefficient (ICC) and 95% confidence internal (CI) were calculated to assess the repeatability between two repeated measurements using OCT-HS100. ICC values between 0.81 and 1.00 indicate almost perfect agreement, values between 0.61 and 0.80 indicate good agreement, and values between 0.41 and 0.60 indicate moderate agreement. ICC values less than 0.40 indicate poor to fair agreement [15, 16].

The strength and direction of linear relationship between OCT-HS100-measured GCC thickness and Cirrus HD-OCT-measured GC-IPL thickness were determined using Pearson's correlation coefficient. Absolute *r* values between 0.68 and 1 indicate strong correlation, values between 0.36 and 0.67 indicate moderate correlation, and values ≤ 0.35 indicate weak correlation [17]. A value of *P* < 0.05 was considered statistically significant. Statistical analyses were performed using SPSS (version 19.0, SPSS Inc., Chicago, IL, USA).

## 3. Results

A total of 92 participants (56 women) were included in our study. The mean ± SD age was 45 ± 14 years. The mean ± SD of ocular characteristics were as follows: BCVA, +0.02 ± 0.04 logMAR; spherical equivalent refraction, −0.98 ± 2.07 diopters; and intraocular pressure, 15 ± 3 mmHg.

Table 1 shows the repeatability of GCC thickness obtained by OCT-HS100. In the subset of 34 participants, the GCC thickness for the overall average and superior and inferior hemifields showed almost perfect agreement with ICC value of 0.992 (95% CI: 0.983–0.996) for the overall average. The high level of agreement was similar for all inner ring subfields and nasal subfields of the outer ring with ICC values ranging from 0.979 (95% CI: 0.958–0.989) to 0.987 (95% CI: 0.956–0.989). The outer inferotemporal subfield also showed almost perfect agreement with ICC value of 0.868 (95% CI: 0.753–0.931). The outer superotemporal subfield had the lowest ICC value of 0.70 (95% CI: 0.481–0.838), indicating good agreement.

Table 2 shows Pearson's correlation coefficient between OCT-HS100-measured GCC thickness and Cirrus HD-OCT-measured GC-IPL thickness. The overall average GCC thickness was strongly correlated with macular average GC-IPL thickness (*r* = 0.83, *P* < 0.001). The corresponding hemifields of each device also showed strong correlations. The strongest correlation was observed between macular GCC thickness and macular GC-IPL thickness (*r* = 0.90, *P* < 0.001).

## 4. Discussion

The present study examined a novel SD-OCT algorithm for the assessment of the macular and perimacular RGCs and demonstrated high repeatability of GCC thickness obtained by OCT-HS100. Furthermore, we showed that RGC parameters between OCT-HS100 and Cirrus HD-OCT were strongly correlated.

We showed excellent repeatability of GCC thickness at the macula, where the anatomical RGCs are multilayered and highly concentrated [18]. This is supported by Tan et al. [8], who demonstrated high ICC values of 0.98 and 0.99 in healthy and glaucomatous eyes, respectively, using RTVue-100. Furthermore, we showed that the repeatability of GCC thickness measurement, specifically at the perimacula, was comparable to that of macular GCC thickness. This may provide a new perspective for RGC evaluation at the course of the arcuate RNFL bundles, which has been shown to be a challenge for current examination techniques. For instance, the use of ophthalmoscopic examination to detect glaucomatous diffuse thinning or wedge-shaped RNFL defect is flawed with substantial interobserver variability [10, 19]. While SD-OCT algorithm-assessed peripapillary RNFL has high repeatability [20], it does not give information on the extensiveness of RNFL defect. RGC damage has been shown to be implicated by retinal hypoxic stress of microvascular lesions that are distributed nonuniformly across the retina [21]. However, an effective technique of localizing this structural RGC damage has not been proven. In this regard, a large area of RGC assessment may provide spatial and morphologic information to evaluate the extensiveness of RNFL defect in addition to quantifiable parameter which is necessary for the staging and monitoring of ophthalmic diseases.

Recent studies had demonstrated that the macular GC-IPL thickness obtained by GCA algorithm, which excludes the RNFL, has high diagnostic ability for early glaucoma [19, 22]. Although the parameter most representative of RGCs in OCT-HS100 is the GCC thickness that includes the RNFL, the diagnostic ability of this algorithm should be further evaluated since our study showed that the GCC thickness encompassing the macula and perimacula was strongly correlated with the macular GC-IPL thickness.

## 5. Conclusions

In conclusion, we demonstrated that the OCT-HS100 Glaucoma 3D algorithm can assess the macular and perimacular RGC parameters with high repeatability. The RGC parameters between Glaucoma 3D and Cirrus HD-OCT GCA algorithms are strongly correlated. A comprehensive assessment of RGCs and their RNFL bundles may be possible with OCT-HS100.

## Figures and Tables

**Table 1:** Repeatability of ganglion cell complex parameters obtained by OCT-HS100 (*n* = 34).

GCC thickness	Mean ± SD (*μ*m)	ICC (95% CI)
Overall	93.96 ± 7.28	0.992 (0.983, 0.996)
Superior hemifield	90.13 ± 7.30	0.984 (0.969, 0.992)
Inferior hemifield	97.82 ± 7.52	0.982 (0.964, 0.991)
Macula		
Inner superonasal subfield	117.72 ± 8.22	0.981 (0.963, 0.991)
Inner inferonasal subfield	116.87 ± 8.42	0.981 (0.963, 0.991)
Inner inferotemporal subfield	105.57 ± 7.79	0.979 (0.958, 0.989)
Inner superotemporal subfield	99.60 ± 7.34	0.980 (0.960, 0.990)
Perimacula		
Outer superonasal subfield	107.40 ± 10.65	0.987 (0.956, 0.989)
Outer inferonasal subfield	118.44 ± 10.59	0.984 (0.968, 0.992)
Outer inferotemporal subfield	79.35 ± 7.32	0.868 (0.753, 0.931)
Outer superotemporal subfield	70.54 ± 6.51	0.700 (0.481, 0.838)

CI: confidence interval; GCC: ganglion cell complex; ICC: intraclass correlation coefficient; SD: standard deviation.

**Table 2:** Pearson's correlation coefficient (*r*) between GCC thickness by OCT-HS100 and GC-IPL thickness by Cirrus HD-OCT (*n* = 92).

Macular GC-IPL thickness by Cirrus HD-OCT^†^	GCC thickness by OCT-HS100			
	Overall average^‡^	Macular average^§^	Superior hemifield^‡^	Inferior hemifield^‡^
Average	0.83	0.90		
Superior hemifield			0.78	
Inferior hemifield				0.82

GCC: ganglion cell complex; GC-IPL: ganglion cell-inner plexiform layer.

All *r* values were statistically significant (*P* < 0.001).

^†^Macular GC-IPL thickness was measured over 4 mm vertical diameter and 4.8 mm horizontal diameter.

^‡^Overall average and superior and inferior hemifields GCC thicknesses were computed over 10 mm in diameter centered on the fovea.

^§^Macular average GCC thickness was measured over 5 mm in diameter.
